# Reconsidering the scribbling stage of drawing: a new perspective on toddlers' representational processes

**DOI:** 10.3389/fpsyg.2015.01227

**Published:** 2015-08-21

**Authors:** Claudio Longobardi, Rocco Quaglia, Nathalie O. Iotti

**Affiliations:** Department of Psychology, University of TurinTurin, Italy

**Keywords:** scribbling, children's drawings, early childhood representation, infant behavior, child art

## Abstract

Although the scribbling stage of drawing has been historically regarded as meaningless and transitional, a sort of prelude to the “actual” drawing phase of childhood, recent studies have begun to re-evaluate this important moment of a child's development and find meaning in what was once considered mere motor activity and nothing more. The present study analyzes scribbling in all its subphases and discovers a clear intention behind young children's gestures. From expressing the dynamic qualities of an object and the child's relationship with it, to gradually reducing itself to a simple contour of a content no more “alive” on the paper, but only in the child's own imagination, we trace the evolution of the line as a tool that toddlers use to communicate feelings and intentions to the world that surrounds them. We will provide a selected number of graphical examples that are representative of our theory. These drawings (13 in total) were extracted from a much wider sample derived from our studies on children's graphical-pictorial abilities, conducted on children aged 0–3 years in various Italian nurseries. Our results appear to indicate that scribbling evolves through a series of stages, and that early graphical activity in children is sparked and maintained by their relationship with their caregivers and the desire to communicate with them.

## Introduction

As children develop, they acquire new meaningful gestures that help them understand and interact with the world that surrounds them. Scribbling is one of these gestures. To see these first traces as a mere consequence of the gesture of drawing or simple hand movement (Burt, 1921; Callaghan, 1999; Dunst and Gorman, 2009) would mean evaluating the graphical product without taking into consideration the level of development of its author. Historically, authors have judged the graphical activity, in the form of scribbles, of children aged 2 or 3 years old, in terms that are exclusively kinesthetic, or of pure motor pleasure, (e.g., Luquet, 1927; Lowenfeld, 1952; Arnheim, 1954; Anning and Ring, 2004; Jolley, 2009; Vinter et al., 2010). However, giving such an exclusive interpretation of this phenomenon means not considering the development that children undergo during their second year of life. The emergence of mental representations and, thus, the ability to use a signifier to evoke meaning, would not seem to be compatible with an activity that stimulates the pleasure of mere exercise. Nonetheless, the vast majority of researchers of child art insists that children are doing nothing more than exercising their limbs. From Luquet (1927), Gardner (1980) and Freeman (1980) we see that there is no talk of actual “drawing” before ages 4–6, when a formal correspondence between the child's traces and the object to be reproduced is recognizable (Malchiodi, 1998; Knight, 2008; Lange-Küttner, 2011). The hypothesis that the reason for which children begin to draw in the first place is their desire for graphical representation or pictorial figuration (Thomas and Silk, 1990; Papandreou, 2014), does not allow researchers to evaluate correctly the meaning of scribbles or to reflect on the reasons that make them such a gratifying activity. The first scribbles appear during the child's second year of life and to not grasp a form of intentionality in their production is the observer's limitation and not the child's. A child's gestures, at age 2, are never mere motor activity; the youngster is able to point, say no with his or her finger, manipulate many objects, grasp things, push them away, hit them, and pummel them as well (Pinto et al., 2011). When the child transfers his or her hand's activities to the line, only *then* he or she draws. Now, the first drawings never have the intention of representing the formal aspects of reality by means of graphical schemas, but, instead, they tell of a world perceived physiognomically by using the line's expressiveness. Starting from our theory, the questions that we asked ourselves were: (a) How does scribbling develop? Does it evolve through different stages, much like drawing does, and can these be classified? (b) What are the various functions that the line carries out during the development of scribbling? With this study, we intend to delineate the first phases of children's drawing through the different meanings that the line acquires progressively, from gesture to representation. We have chosen to do so by reporting a number of suggestive examples (13) that we have drawn from a much wider sample derived from our studies (Longobardi et al., 2012; Quaglia et al., 2015) on children's graphical-pictorial abilities, conducted on children ages 0–3 in various nurseries, located in one of Western Italy's biggest cities. We shall present said examples along with our theory, as we advance through its various stages.

## The line as gesture

### The birth of the line

The child does not discover the line in a fortuitous manner. Adults always tend to attribute children their own ways of thinking and, consequently, believe they share their same joy of discovery; but what excites children is not discovery in itself, it is discovering they are able to do things.

When we have talked about the beginning of graphical activity, we have often underlined the child's apparent lack of interest for his or her creations (Thomas and Silk, 1990; Ring, 2006) and this has made us believe that children draw mostly for the satisfaction they receive from the mere motor activity of this gesture. In our experience as a researchers of child art (Quaglia and Saglione, 1976; Longobardi et al., 2012.; Quaglia et al., 2015), we have discovered that the child's first graphical gestures are not motivated by the graphical product, but by the desire to imitate adults, particularly parents, and teachers. In fact, they imitate the gesture and not the result. To behave *like* an adult is the child's most primitive and intense source of joy. His or her desire is to receive the adult's attention, firstly for what he or she can do, and secondly for what he or she has done. The interest toward his or her artistic creations begins once the child has moved from *acting* like adults, to *doing* what adults do, while striving for better oculomotor control. We can then begin to notice a trace that mimics writing, accompanied by the child's statement that he or she has “written.” The act of learning is never casual, but it always takes place inside a meaningful relationship. No activity is relevant in itself, but it acquires relevance when it becomes the symbol of a relationship.

Our theory is coherent with Vygotsky's (1978) ideas on learning through imitation, and Bruner's (1983) social-constructivist perspective. Children, aided by adults, progress inside their own Zone of Proximal Development (Vygotsky, 1978) and gradually begin to master abilities that had not been developed until that moment, abilities such as scribbling. The adult's role in this process is vital: it is only through the meaningful relationship that the child has established with his or her caregiver that graphical abilities can flourish and develop completely.

### Good traces and bad traces

In the study conducted with Stefano (Quaglia and Saglione, 1976), researchers carefully avoided teaching him how to draw real objects and left him with the maximum liberty of expression and observed the “spontaneous” evolution of the line. The employment of the line from “affective gesture” to “contour of a picture” measures not just the evolution of motor and perceptive functions (Widlöcher, 1965; Matthews, 2003; Lange-Küttner, 2012, 2013), but also reflects the evolution of the dynamic character of primitive perception in particular (Werner, 1940; Wood et al., 1976; Morra, 2002).

For the child, the objects of the external world are not just geometric figures: they are also elements of dynamic events. Children have little interest for the static qualities of objects, but they are quite fascinated by their dynamic properties. At this developmental stage, a dog is not something that possesses and objective form and a mixture of parts, but it is “something that bites or barks” (Gantshewa in Werner, 1940, p. 70). As Worthington, along with other researchers, tells us: “Children explore personal meanings through their free drawing” (Worthington, 2009, p. 38). In other words, the objects of the external world can be desirable or scary, good, or bad, and they are experienced mostly through the affective and motor behaviors of the subject. Child animism is children's tendency to perceive things as living and endowed with intentionality (Piaget, 1964; Morra, 2002). The line is filled with the intentionality of the gesture and it initially translates the affective qualities of the objects through its physiognomical characteristics.

Therefore, alongside the semantical gestures of Yes and No, as described by Spitz (1957), we witness the appearance of a second dyad of gestures: caressing and hitting. Children caress what they like and hit what hurts them. In these new gestures, we discover the cause of the creation of the line.

Young Stefano (aged 18 months), after having discovered scribble-writing, began to develop an interest in books, writing over them, or, better yet, re-writing them. In the attempt to save the books, his parents bought him books that were appropriate for his age and full of pictures. He immediately began to scribble over them, but the line did not resemble writing anymore, instead it began to show two distinct formal organizations: a soft, round line (good line) and a thick, broken line (bad line). Fascinated by the pictures he was observing, Stefano desperately tried to grasp them with his hand, scratching them; then, he resorted to the aid of the line, scribbling over them and, when he was done, he happily stated: “mine!,” as if he had somehow taken possession of the picture. What he could not grasp with his hand, he had grasped with the aid of the line, intangible as the picture he wanted to touch. When faced with pictures that were scary, instead, the child would hit them violently with the tip of the pencil or rub them until they disappeared.

The act of scratching to grasp and hitting with the hand, had thus been substituted with a graphical behavior that had the same purposes. The line, as an extension of the hand, expresses its same affective meanings and intentions.

**Figure 1:**
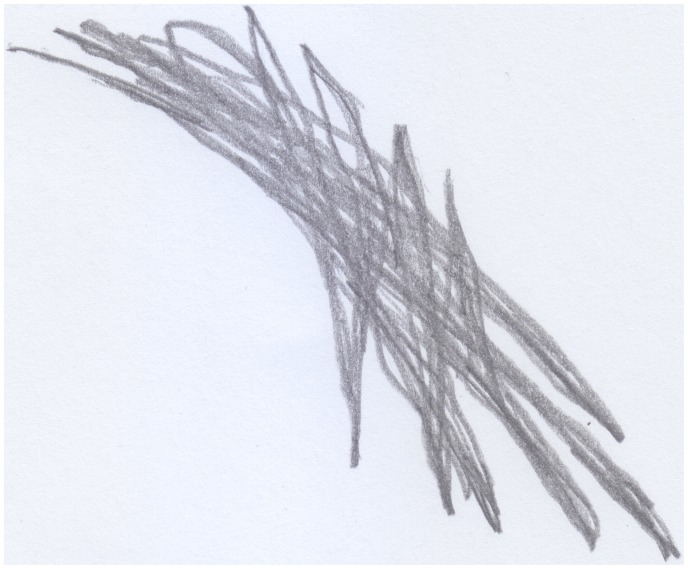
**Stefano's (18 months old) drawing of the table he hit his head against; an example of “bad” trace**.

**Figure 2:**
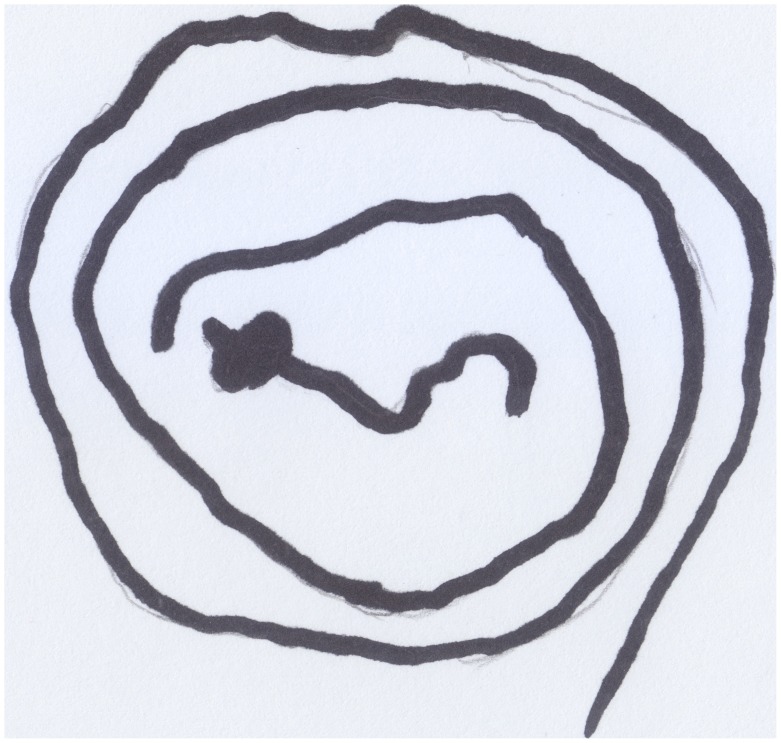
**Stefano's (18 months old) drawing of his mother; an example of “good” trace**.

After Stefano had hit his head against the corner of the table, and after he had avenged himself by hitting the table, he made a drawing of it; that is, he produced a series of thick, superimposed, vertical lines (see Figure 1). Stefano had no intention of representing the table in its physical-geometrical form, but he wanted to represent the act of hitting an object he considered “bad.” The line would instead become round and soft when the youngster would state he had made a drawing of his mother (see Figure 2).

**Figure 3:**
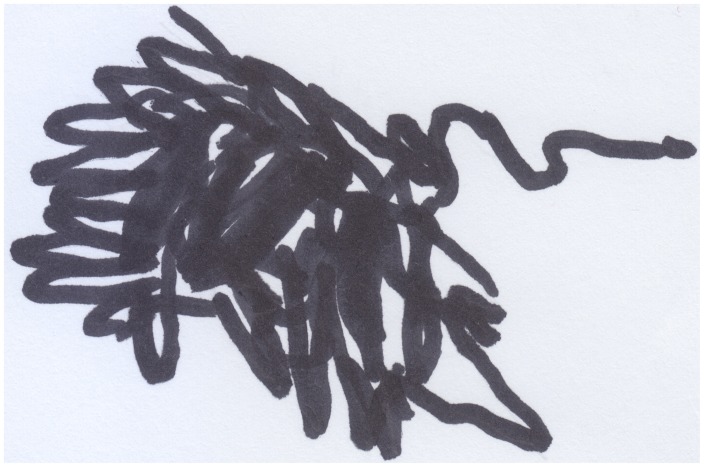
**Marco's (24 months old) drawing of a “bad” dog, presented to his mother while saying: “it barks!**.”

Marco (aged 24 months) had been scared by a big dog during a walk with his mother; when he arrived home, he drew a thick, messy line, and showed it to his mother saying: “it barks!” (see Figure 3). By using the line, the child managed to represent the dynamical aspects of the dog and control a reality that he found menacing. We witness this same kind of intention in Matthews' (1998 in Matthews, 2003, p. 92) description of a 3-year-old Chinese Singaporean boy, Evan, who was scared by a sudden crash of thunder and made “sudden push-pull actions with his pencil pressed hard against the paper,” we believe that this was an attempt, on the child's part, to give meaning to what had just scared him, and it allowed him to represent this phenomenon on a plane that he could understand and control to his liking.

The line, when perceived physiognomically as the act of caressing or hitting, ideally represents the child's relationship with events or objects of the external world through two types of traces: good traces, round, and soft, and bad traces, heavy, and disorganized.

In short, the child's entire graphical production initially presents this double formal aspect, and where we, as observers, *see* vertical, horizontal, ovoid, oblique, or spiral lines (Kellogg, 1979; Uttal et al., 2006), the child *feels* good, happy, playful, or bad, ugly, scary, and sad lines. And since reality can be defined essentially as good or bad, the use of good and bad traces, exhausts all of the child's representative necessities. Such traces and line styles have a universal nature (Golomb, 2002) and belong to culturally inherited symbolic systems that children naturally learn to use for their own expression (e.g., zigzags as “bad” lines).

At the beginning of the drawing experience, children have no desire to reproduce the formal qualities of objects, but the positive or negative experiences that stem from the encounter with the objects of reality.

Summing up, in the affective relationship with the outside world, the child notices correspondences between the dynamical properties of such objects and his or her internal statuses of emotional evaluation. Graphical activity, like any other play activity in general, allows the child to mediate some important affective relation between internal and external world, reproducing them in an area in which reality may be corrected and controlled. When the mediation attempt succeeds, we can notice a certain degree of satisfaction in the child. The criteria for success reside in the physiognomic characters of the child's perception, for which the lines come to life and act upon the objects, representing them affectively.

### Use of scribbling for representing actions and relationships

Graphical activity absolves many functions from the start, one of which is play.

**Figure 4:**
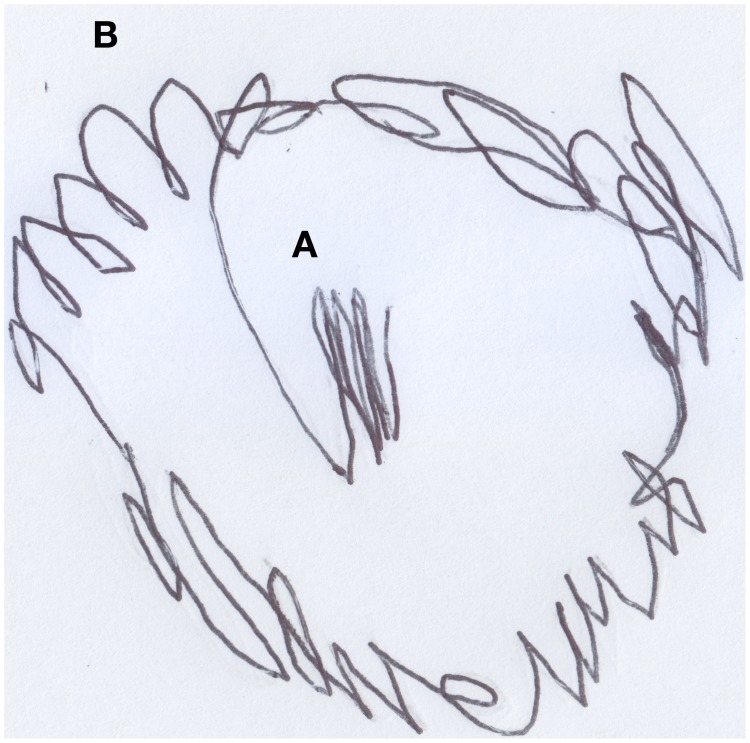
**Anna (33 months old) playing with stickers, a “good” scribble**.

Anna (aged 33 months) loved to play with stickers. In a moment of pause from her game, the child was asked to draw “Anna playing with stickers” (see Figure 4). Initially, the child traced a couple of vertical lines in the middle of the sheet of paper (see Figure 4A), then she scribbled with great certainty a curlicue around the first trace (see Figure 4B). She was then asked to point out where she was located inside the drawing. Without hesitation, she indicated the vertical lines in the middle of the drawing. When asked “and what are these other lines?” she replied: “it's Anna playing with stickers.”

**Figure 5:**
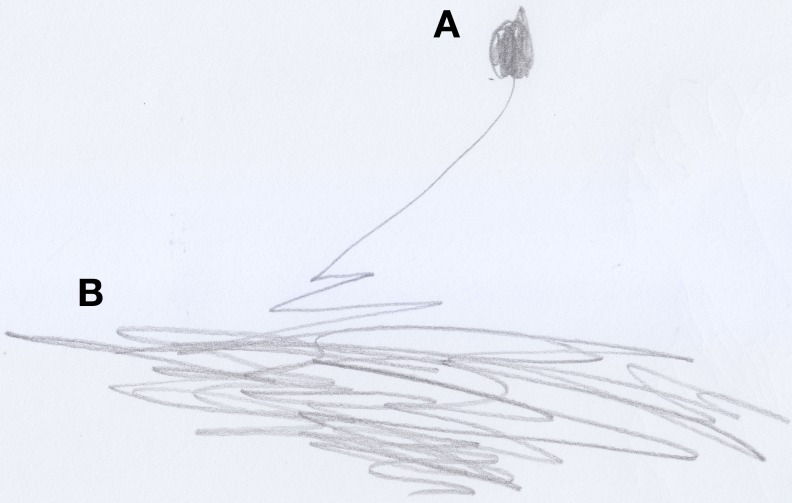
**Anna's (33 months old) drawing of Enzo hitting another child, a “bad” scribble**.

After finishing the drawing, the child was asked to make another drawing, this time of Enzo, the classmate she disliked most because, according to her, he was mean. Anna did not draw this picture happily (see Figure 5); first she traced a tangle of lines (see Figure 5A), and then she sketched other lines in the bottom part of the sheet of paper (see Figure 5B), without putting much care or effort into them. When asked: “Where is Enzo?” she pointed out the tangled lines in the center of the picture. Next, she was asked about the other lines in the drawing and the child answered that those lines were: “Enzo hitting another child.”

In both drawings we notice cores of scribbles, that represent the subjects, from which lines are spawned and would represent, in the first case, Anna at play (round and soft line) and, in the second case, Enzo “running around, hitting and being mean to his other classmates” (decisively heavy and broken line).

**Figure 6:**
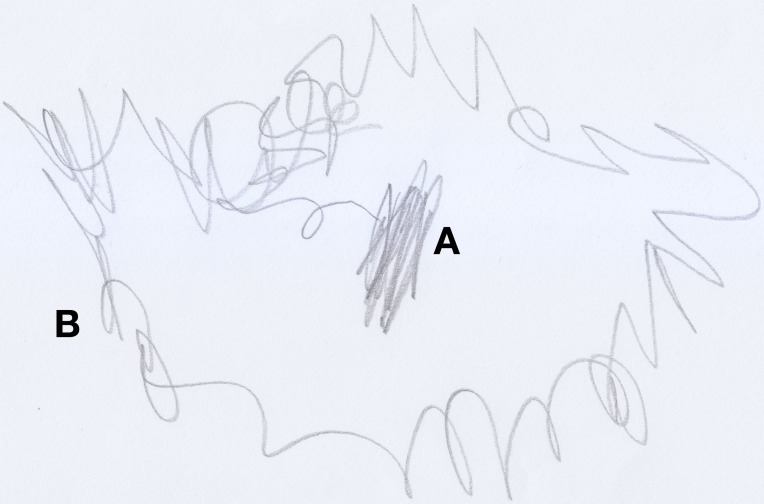
**Michele (32 months old) playing ***scatolino***, a positive self-portrait**.

**Figure 7:**
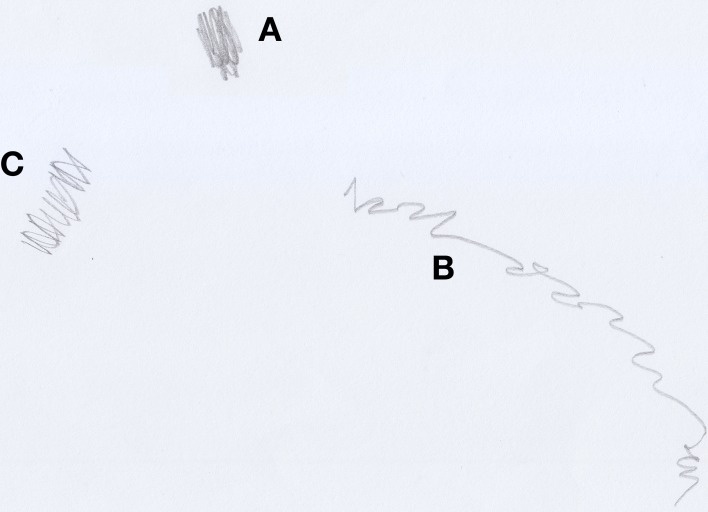
**Michele's (32 months old) drawing of Luca, the “bad” child who always steal his pencils, a negative scribble**.

Michele (aged 32 months), when asked to make a drawing of himself playing (see Figure 6), traced, at first, some broken lines in the center of the sheet of paper (see Figure 6A) and then, using a lighter, rounder line, expanded his scribble (see Figure 6B). When asked: “where is Michele?” the child grasped the researcher's finger and passed it first over the line in Figure 6A and, subsequently, over the entire scribble, specifying that he was playing “*scatolino*,” a game which involves a small box. After having identified a “bad” child along with the subject, Michele drew the picture of Luca, the mean boy that always stole his pencils (see Figure 7). He drew three scribbles on the same sheet of paper in rapid succession: the first one (see Figure 7A) represented Luca, the second one (see Figure 7B) represented Luca playing with Michele, and the third one (see Figure 7C) would, instead, be Luca stealing Michele's pencils.

In this example, we can distinguish three different types of scribbles, each one representing the same person in three different moments. In Michele's drawings we can easily see which traces indicate a pleasant activity (see Figure 7B), and which indicate an unpleasant one (see Figure 7C).

**Figure 8:**
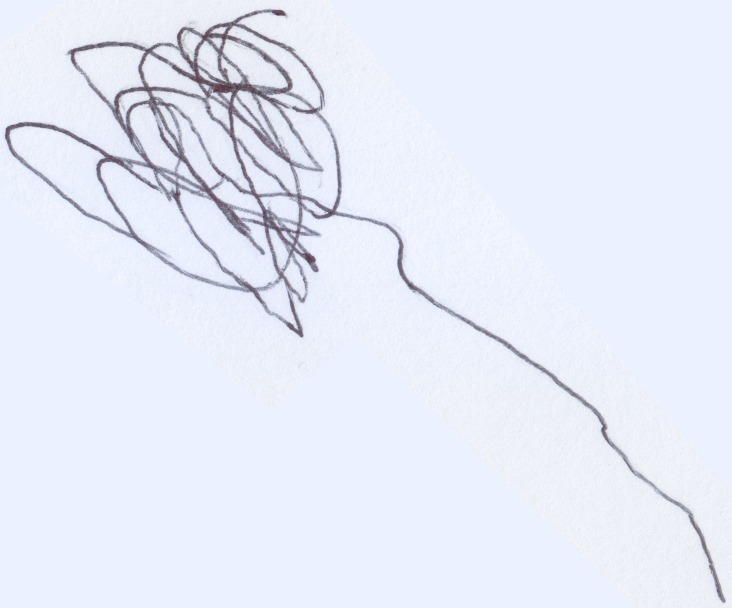
**Elena's (34 months old) self-portrait, a “good” scribble**.

**Figure 9:**
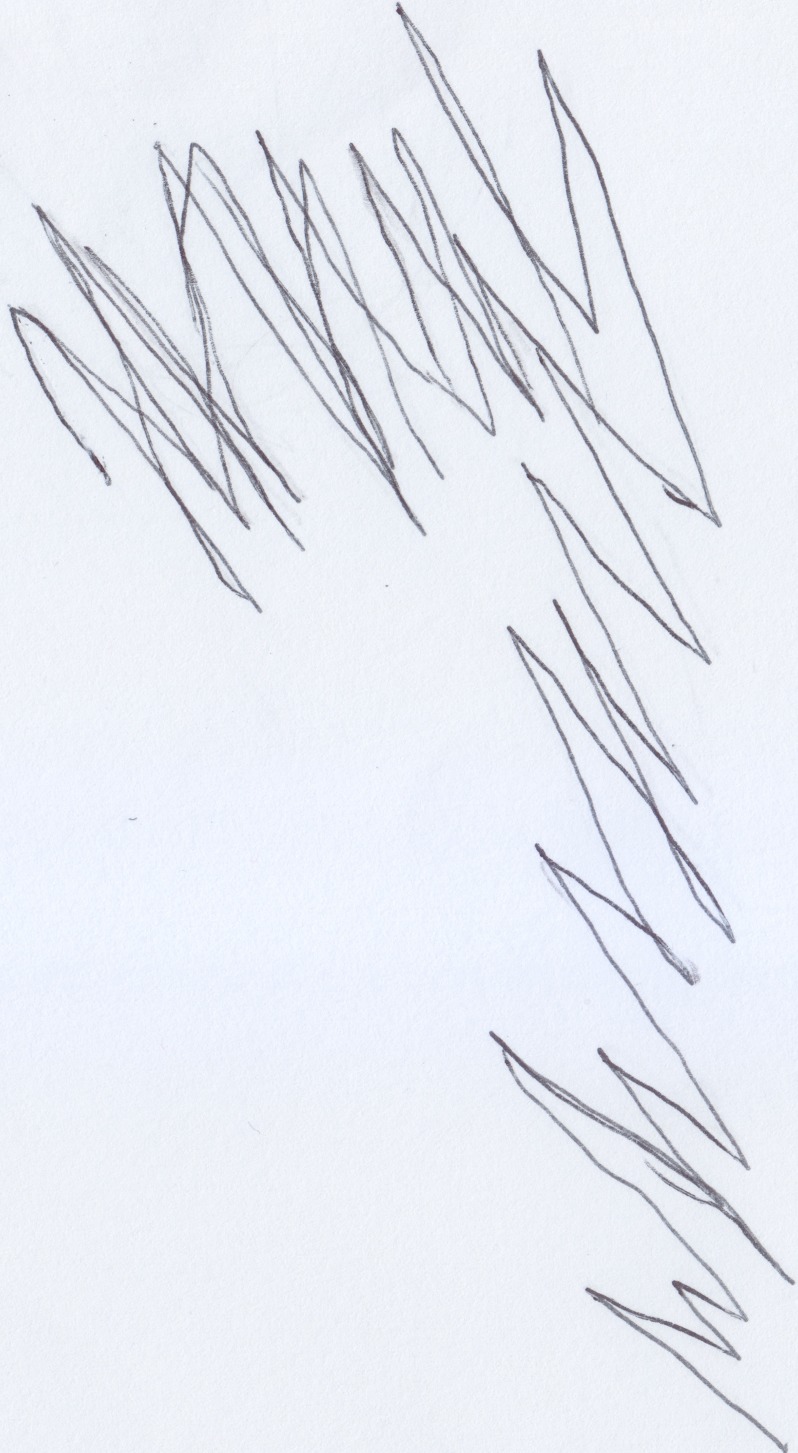
**Elena's (34 months old) drawing of Fabio pushing other children, a “bad” scribble**.

In Elena's drawing (aged 34 months), we can observe in a much clearer manner what has been stated up to this point. Notable differences can be seen between the scribble with which the child reproduces herself (see Figure 8) and the one with which she reproduces Fabio, a child that is “mean to her and pushes her” (see Figure 9). The trace that symbolizes Elena, curvy, and soft, represents a “pleasant play situation,” while the trace symbolizing Fabio, marked, and pointy, ideally represents him during the act of “pushing.”

These two kinds of traces, round in one case and broken in another, not only express an action *per se*, but most of all they express the good or bad quality of the action in itself.

In these types of scribbles (Longobardi et al., 2012; Quaglia et al., 2015) children express themselves through the line's movement or, better yet, with the characteristics of such movement, as they associate it to people and actions that are qualitatively different. The sheet of paper, thus, becomes a play area and the line is the instrument that animates the child's characters and fantasies. The process of naming a scribble does not indicate the child's desire to represent reality or his or her recognition of some sort of similarity between the drawing and a random object, but, instead, it would simply indicate that the scribbles have become the symbolical witnesses of experiences with objects experimented by the child, mostly through an affective behavior. This would also mean that children alter the line's quality, color, and shape to express their feelings about certain topics, and that these vary depending on whether they regard them as positive or negative (Burkitt et al., 2009).

## The line as movement

The physical-geometrical qualities of reality are knowledge's final form: as Piaget (1964, 1967) tells us, all of the newborn's initial behavior can be defined by saying that he views the world as a reality to be discovered through tasting and suckling (Anning and Ring, 2004).

Subsequently, after having gained an erect stance, children become more and more interested in the objects that surround them. This is Spitz's “No” phase, or Margaret Mahler's (Mahler et al., 1975) *Rapprochement* subfase (between 15 and 18 months): the child discovers the perils of the world. People, objects, and experiences tend to be either “good” or “bad.”

It is not just the affective behavior that influences children's manner of experiencing objects, but also their own motor behavior helps in determining a dynamical perception of reality (Werner and Kaplan, 1984; Ebersbach et al., 2011; Lange-Küttner, 2014). On the other hand, children are naturally immersed in a world that moves and makes noises and we can notice this fascination with the dynamical properties of objects in their graphical activity, most of all in the *onomatopoeic scribble*, which is any trace that is accompanied, during its creation, by an onomatopoeic expression.

**Figure 10:**
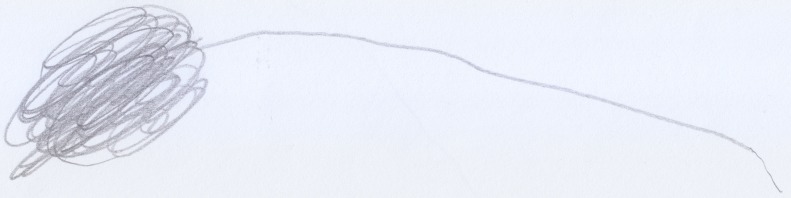
**Stefano's (18 months old) first onomatopoeic scribble, the motorcycle**.

Stefano (aged 18 months), always had a particular love for any kind of vehicle, most of all motorcycles, and whenever he saw one he would signal its presence with the characteristic sound “Vroom, vroom.” He would repeat this noise when he played with his toy cars and, finally, he added this sound to his scribbling activity (Quaglia and Saglione, 1976). On such occasions, he enjoyed drawing a line that expanded over the floor and stretched through the whole apartment. When asked what he had drawn, he would repeatedly state that he had drawn a “Bibì” (i.e., “beep beep”), a term with which he indicated both cars and motorcycles indiscriminately. When he was finally convinced to draw on sheets of paper, he created the following drawing (see Figure 10): a line that, not being able to expand endlessly, reduced itself to a tangle of “streets.”

Although he was not able to produce a figurative drawing, Stefano had imagined a moving object and had graphically represented precisely the movement associated with this particular sound.

Another example of onomatopoeic scribbling can be found in Matthews' recount of Evan, the boy that had scribbled after having been scared by lightning, and his behavior while drawing: “As Evan makes the push-pull to describe the sudden discharge of lightning, he says, “Aaaaaaaa…,” and raises his pencil high above his head. Then, with an overarm action, aims the pencil down into the pencil box on the table. He says “Aaaaaa…” as his moving hand describes a descending arc in space and, “Bfff!” at its moment of impact, after which he lets the pencil go.” (Matthews, 2003, p. 93), or Li Yu's drawing of the “Queen of Snakes” gliding out of a tunnel, made while producing hissing sounds accompanying the gliding movement being registered on the paper.

Certainly, the onomatopoeic scribble does not exhibit any qualities that qualify it as a real drawing, but it can, in fact, become so, if we assess it while referring to the child's representative goal. We can no longer accept the hypothesis that scribbles are a mere motor expression and, at the same time, we cannot support the idea that no form of representation is possible without figurative schemes.

With onomatopoeic scribbling, children conclude a very important part of their graphic development because the line goes from representing their fantasies to progressively assuming the shape of the objects which it depicts (Quaglia and Saglione, 1976). Now, in the measure with which the objects take form on the sheet of paper, they lose their real speed, symbolized by the movement of the line, and they acquire it in the realm of pure imagination. In other words, the line visibly symbolizes the actions performed by the imaginary objects; in the schematic drawing, instead, the represented objects are sufficient to testify the actions they now perform only inside the child's own imagination.

## The line as contour

From the onomatopoeic scribble onwards, scribbles seem to organize themselves progressively around a “scheme embryo” or “fundamental graphical nucleus” (Quaglia and Saglione, 1976).

It is difficult to say what might motivate the child to abandon the “scribble line” in favor of the “drawing line,” which will gradually become a line of contour and nothing more. The artist's emotive states, after the acquisition of a figurative schema, will reveal themselves in the subjects of his or her drawings: objects that are loved or feared.

According to Kellogg (1979), pre-scholastic and scholastic institutions are responsible for imposing on children the reproduction of real objects, and this would determine the end of child art and the beginning of a graphical activity that no longer supports the child's expressive needs (Einarsdottir et al., 2009). Luquet (1927), instead, believes that children casually begin to notice some analogies between their chaotic productions and real objects. Freeman (1980) has a similar stance on this argument, stating that children begin to reproduce reality spontaneously, since their sole motivation for drawing can be found in the production of graphical or pictorial representations. In fact, most researchers believe that the child is naturally inclined to draw symbols that mostly refer to visual experiences (Arnheim, 1954; Selfe, 1983; Lange-Küttner, 2008), and that there can be no talk of real drawing until the beginning of the reproduction of figurative schemes that can be easily identified with real objects (Thomas and Silk, 1990; Malchiodi, 1998; Lange-Küttner et al., 2014). Actually, as we are trying to demonstrate, it is not the representative intent that favors the birth of figurative drawing, but the gradual passage from the objects' dynamical properties to their formal qualities. It is the syncretic nature of primitive perception that slowly disappears and movement alone is no longer sufficient to continue the “dialog” with the objects. Children that seek or find analogies between their graphical product and a real object, reveal a new level of organization of their cognitive development and this is not a casual or fortuitous event.

**Figure 11:**
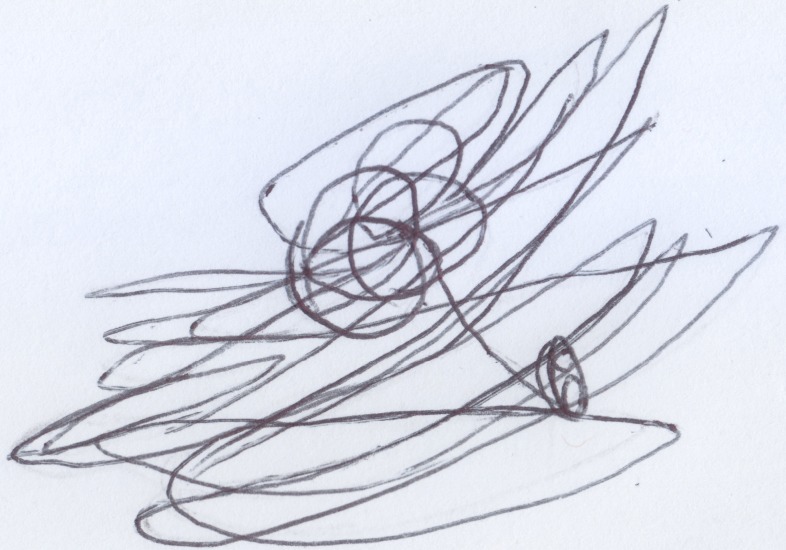
**First dynamic nucleus of a motorcycle, a “good” scribble**.

**Figure 12:**
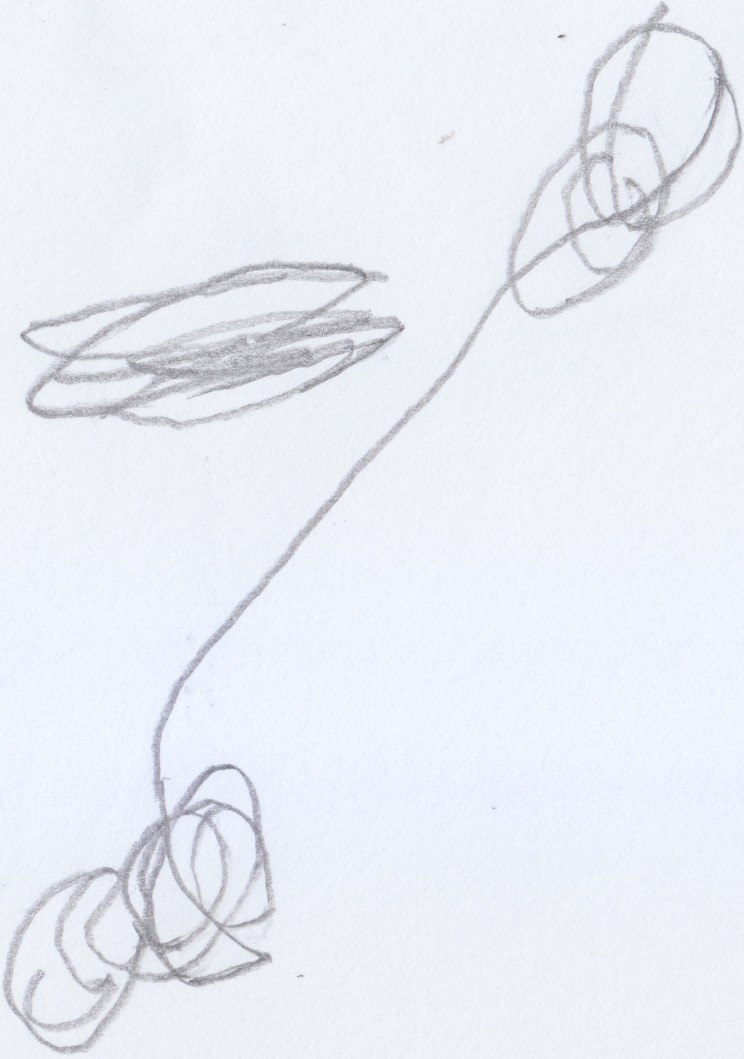
**Beginning of fixation of the dynamical characteristic of the object in specific parts (i.e., the wheels)**.

**Figure 13:**
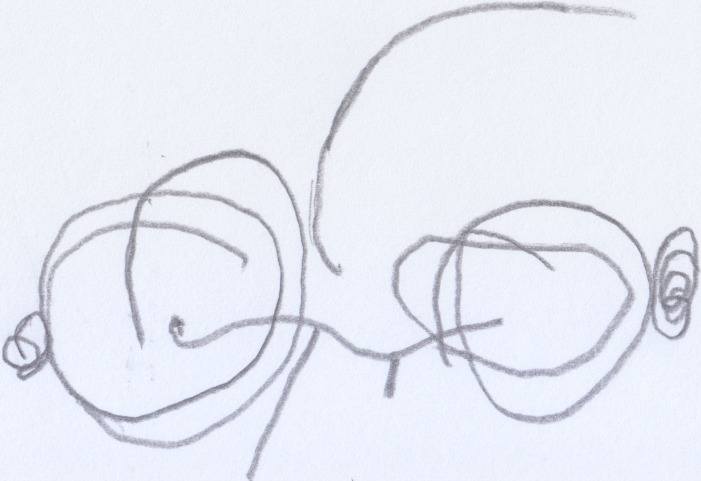
**The motorcycle as a primitive figurative drawing, containing all the elements that are necessary for its recognition**.

Young Stefano (aged 18 months), began to draw two scribbles connected by a line (see Figure 11), demonstrating that he had acquired a new conscience of the properties possessed by the object he was fantasizing about (i.e., the motorcycle). After a series of drawings that became increasingly more articulated and detailed, he created the first picture in which the primitive scheme of the motorcycle can be clearly noticed (see Figure 12). In the picture we can see the tires, the lights, the handlebar, the kickstand, and a first line of the chassis (see Figure 13).

It is our opinion that children spontaneously abandon the scribble, which is a dynamical representation of an object, in favor of figurative schemes, that better suit their new creative needs to no longer just represent reality, but to make reality “real.”

Scribbling can be compared to a sort of “graphical monolog” that accompanies and reinforces the child's imagination; its function is similar to that of egocentric language. Furthermore, we can notice important parallel transformations between the development of drawing skills and language development (Vinter et al., 2013). Thus, we can witness a reorganization of both the child's language system, which allows for better communication effectiveness, and graphical system, with the appearance of figurative schemes (Malchiodi, 1998; Jolley, 2009). In both cases, owing to the child's improved intellectual abilities, we notice the emergence of behaviors that are more adequate to the comprehension of the person receiving the message, be it spoken, or graphic. Scribbling, as movement, represents actions in the form of verbs; now, with figurative drawing, it is as if the child had added the subject of the action to his graphical discourse.

Nonetheless, the objects' dynamical elements or aspects do not disappear, instead their presence gives them function.

In the whole figurative period of child art, movement is represented through a particular perspective that is labeled “dramatic.” The form or dramatic representation of an object is what suggests the action that subject and object can perform. Children do not want to communicate an object's structural information (Freeman, 1980), through a canonical perspective (Hochberg, 1978, 1985; Anning and Ring, 2004), they just want to make their drawing meaningful.

Therefore, the house is shown frontally not because the child wants to give the observer the highest possible number of structural information, but instead because the door suggests the artist the idea of entering and exiting. In an experiment (Longobardi et al., 2012), we showed children aged 6–8 years old the drawing of a house in frontal vision, but left out the door. The children stated that that could not be the drawing of a house because you could not go inside or exit from it. At the observer's objection that it could be the back of a house, the children answered that it looked like a prison because you could not enter it or leave it. And so, the handle of a mug, for Freeman and Janikoun (1972), is drawn to make the object recognizable, but for the child, a cup is not an object you observe, it is something you drink from, and to do so, you have to draw the handle.

## Conclusions

Therefore, going back to the research questions that we asked ourselves at the beginning of this paper, namely: (a) How does scribbling develop? Does it evolve through different stages, much like drawing does, and can these be classified? (b) What are the various functions that the line carries out during the development of scribbling? We believe that we have found our answers. Scribbling evolves through various stages, sparked, and maintained by children's relationship with their caregivers, and by their desire to communicate with them. We have traced and classified these steps (Longobardi et al., 2012; Quaglia et al., 2015), observing the evolution of the line as it gradually acquires complexity, and it shifts from being the media through which the action is represented physically, to becoming a mere contour, a hint, of actions that are no longer present on the paper, but have become abstract and have been transferred into the child's own imagination.

According to our perspective, children are not interested in representing reality at any time of their graphical development; they do not draw what they know about reality (Luquet, 1927), or what they see (Arnheim, 1954), they choose to draw experiences. The first traces are graphical representations of gestures; onomatopoeic scribbles are kinesthetic translations of creative fantasies; figurative schemes, developed from the need to identify reality, are a scenic apparatus that has been set up for a fantastical representation. The child that draws a plane or a ship is actually drawing an adventure that develops as the drawing proceeds; in every drawing there is a story or a script that constitutes the most rewarding side of child graphical behavior. We believe that this new interpretation of scribbling could lend itself to new studies and offers research opportunities for a better understanding of early child development, while creating new applicational and interpretational spaces for children's graphicacy in general.

As we have already suggested in previous studies (Quaglia et al., 2015), we believe that our findings might aid researchers and clinicians in investigating toddlers' representations of their relationships with the reality that surrounds them. Our theory could be the starting point for the development of better and more precise assessment tools that exploit the link between scribbles and the child's own perception of the external world, in order to identify early markers of child distress or other emotional manifestations. This would be a positive aspect because it would provide professionals with an additional, and virtually cost-free, tool that can be used to gather further information on children and their own internal world, at least a year in advance as compared to classical graphical tests, which are not usually administered before age four.

### Conflict of interest statement

The authors declare that the research was conducted in the absence of any commercial or financial relationships that could be construed as a potential conflict of interest.
